# Sexism, attitudes, and behaviors towards violence against women in medical emergency services workers in Erzurum, Turkey

**DOI:** 10.1080/16549716.2018.1524541

**Published:** 2018-10-01

**Authors:** Elif Okşan Çalıkoglu, Aysun Aras, Maysa Hamza, Ayşegül Aydin, Onur Nacakgedigi, Patrick Marius Koga

**Affiliations:** a School of Medicine, Department of Public Health, Ataturk University, Erzurum, Turkey; b Davis, School of Medicine, Department of Public Health Sciences, University of California, Davis, CA, USA

**Keywords:** Violence against women, health workers, medical emergency services, gender bias, archaic sexist attitudes, Turkey

## Abstract

**Background:** In Turkey, almost every 4 out of 10 married women have been subjected to physical abuse by their spouses. Although studies on the prevalence of domestic violence in Turkey abound, little has been published about first responders’ attitudes and behaviors towards violence against women and on sexism.

**Objective:** Our study examined the attitudes and behaviors of Erzurum City medical emergency services workers towards violence against women, and their relationship with sexist attitudes.

**Methods:** A cross-sectional survey was conducted among 370 medical emergency service personnel using a self-administered questionnaire of 35 items, which included two scales utilizing a three-point Likert format; 15 questions measured attitudes and behaviors towards violence against women (VAW) and 12 items measured sexist attitudes.

**Results:** The mean age of participants was 29.6 ± 8.0 years with a sex distribution of (47.6%) women and 194 (52.4%) men. Less than half of the participants (48.5%; n = 173) felt competent in recognizing and managing VAW; moreover, when faced with such cases, 18.5% (n = 67) said they would try to reconcile the victim with the perpetrator. Male participants had higher mean scores both on VAW (20.7 ± 5.2 vs. 16.9 ± 2.8; t = 7.927; p < 0.001) and on sexist attitudes (24.3 ± 5.3 vs. 18.6 ± 4.3; t = 1.714; p < 0.001). Age (B = 0.067; 95% CI: 0.014–0.119; p = 0.013) and sexism scores (B = 0.487; 95% CI: 0.407–0.566; p < 0.001) were revealed as independent significant predictors of the VAW attitude scores.

**Conclusions:** Educational and public health measures must be instituted to change attitudes and behaviors towards violence against women; measures must focus not only on violence but also on sexism. Health care professionals need to reflect on their own gender biases in clinical practice and prevent gender discrimination.

## Background

Worldwide, whether with or without partners, 35% of women experience physical and/or sexual violence throughout their lives [1]. Statistics about domestic abuse in Turkey are no less alarming. The findings of the most comprehensive Turkish survey to date on this topic, the 2014 ‘National Research on Domestic Violence Against Women in Turkey’ [2] in its sample of 12,725 women aged 15–59, show not only the magnitude of the problem but also the subsequent burden of mental disorders following the violence such as posttraumatic stress disorder, depression, anxiety, sleep and eating disorders, alcohol and drug abuse, and suicidal behavior. Given the alarming global health burden of 9.5 million disability adjusted life years (DALYs) due to the mental health consequences of violence against women in their reproductive age group worldwide [2], future studies in Turkey should inform on the issue of burden per specific disorders both first responders and policy makers. 39% of the married women in Turkey declared that they were subjected to physical abuse at any stage of their lives; in other words, almost every 4 out of 10 married women have been subjected to physical violence by their spouses/partners [3].

Domestic violence leads to a deterioration of the general health status of the victims [4] and their utilization of health services increases [5]. There is a fourfold risk for mental disorders in women who have experienced physical and/or sexual violence; 12% of victims reported at least one suicide attempt as a result of the brutality perpetrated on them [3]. As health institutions have important roles and responsibilities concerning this public health problem, they are essential stakeholders in the campaign against violence. Among them, first responders such as emergency services are of the first order of importance both to victims and to the prevention campaigns [6]. While 30% of women seeking help at emergency services do so because of the violence they have experienced [4], this rate can increase substantially when psychological violence is added [7]. One study exploring patterns of help-seeking behaviors among violence victims, and the attitudes of health care personnel vis-a-vis violence, show that less than half of the women receiving medical treatment for injuries reported their abusers, or were asked about the context of their injuries [8].

Sexist attitudes have been claimed to play an influential role in the acceptability of intimate partner violence [9]. For instance, it has been found that respondents who endorse sexist attitudes also report more accepting attitudes towards intimate partner violence (IPV) [10].

Laws and regulations requiring identification of cases of violence against women (VAW) and proper reporting are often a thin veneer on the substantially different bedrock of traditional social norms developed in and suited for past times. The Article 280 of the new Turkish Criminal Code mandates health care personnel to detect and report cases of domestic violence; the failure to do so is a criminal offense [11]. Moreover, most Turkish women report their husbands as the perpetrators of the violence they regularly experience [12]. It is therefore essential for those working in health care institutions to be able to recognize violence against women and to undertake proper interventions.

In 2002, the Emergency Department of Ege University Hospital, one of the most critical health service provider and referral center in Western Anatolia, conducted a cross-sectional study on the knowledge and behaviors of its doctors and nurses about intimate partner violence with astounding findings. At least one reason to justify physical violence against women was accepted by 69.0% of the female and 84.7% of the male participants [13].

### Objectives

We hypothesized that the highest levels of acceptance of VAW would be found among those participants who also demonstrated sexist attitudes. Hence, our aim in this study was to examine the attitudes and behaviors of medical emergency service workers towards violence against women and to look for a possible relationship with sexist attitudes.

## Methods

### Study design and setting

Our study conducted in the spring of 2016 in Erzurum City, Turkey, adopted a cross-sectional survey design. The people living in this large Eastern Anatolia city of 750,000 inhabitants, are known for their traditional, very conservative lifestyle. In this patriarchal region early marriage and polygyny are still prevalent; forced marriages still take place and arranged marriages are still the norm, although an educated minority of young women are increasingly tolerated to choose their partners [14]. Research permission was obtained on 19 July 2016, from the Turkish Ministry of Health, Medicines and Medical Devices Authority (Issue No. 93189304-514.99-90759); written consent from the relevant institutions and verbal approval from the participants were secured before conducting this research.

### Participants ‎

The eligible universe of the study consisted of 460 medical emergency health personnel (physicians, nurses, emergency medical technicians, and paramedics) working in Erzurum City, attached to emergency hospitals, university hospital’s emergency room, and the city’s three main family health centers. An invitation letter to participate in this study was delivered by mail to all 460 health workers. The letters were sent via the official mailing system of the local health directorate. The letter was followed a week later by a telephone call to reiterate the invitation. Some of the ambulance workers could not be reached because they were on route at the time of the call; others stated they did not want to participate in the study. Eventually, 370 of the 460 health workers participated in our study giving a response rate of 80%. The procedure was explained in detail to all 370 participants before the commencement of the survey, and the confidentiality of individual participants was strictly protected. No participant names were registered and also no incentives were offered to participants.

### Variables

The main outcome of the study was a sexist attitude score obtained via ‎a 12-item questionnaire. As secondary outcome measure of the study, we collected data on VAW attitudes of the participants‎. Building on previous work conducted in Turkey [13], a vigorous literature search and expert opinions served as a basis for the development of the survey instruments. After an expert panel, ‎including four specialists of public health, three public health nurses, one social worker, and one ‎psychiatrist, established content validity, four focus group discussions from the target population with six participants in each group were conducted for face validity. Additional items, items needed to be modified/removed, intelligibility, grammar ‎and spelling‎ were also discussed in detail. The responses to the attitude questions were graded on a three-point Likert scale (1-Agree, 2-Neither agree nor disagree, and 3-Disagree). Three of the VAW questions (‘VAW is a public health problem’, ‘VAW is disturbing to me’, and ‘I am disturbed by the news of women homicide in the media’‎) and two of the sexist attitude questions (‘I believe in gender equity’ and ‘A woman should be free to spend her money as she wishes’‎) were reverse coded. Scores for VAW and sexist attitudes were calculated for each participant by adding the responses together. Psychometric properties of the VAW and sexist attitudes questionnaires were as follows: The 15-item VAW Attitudes Questionnaire had high internal reliability with a Cronbach alpha value of 0.835. Principal component analysis with Varimax rotation revealed four factors explaining 56.6% of the total variance with the following subdomains: Feelings, Justification (Physical Violence), Justification (Sexual violence), and General/cultural. The final version of the data collection tool consisted of 35 items: Four questions collecting demographic information, one question asking for recent experience in VAW, one question asking about the action in case of facing VAW, two questions on sexual preference, 15 items querying VAW attitudes, and 12 items querying sexist attitudes. Answering the survey questions took less than 10 minutes.

The 12-item Sexist Attitudes Questionnaire too had high internal reliability with a Cronbach alpha value of 0.838. Here the principal component analysis with Varimax rotation revealed two domains (Family/Household and Work/Social Life) explaining 47.6% of the total variance.

### Statistical methods

The data collection tool was administered in March-April 2016 to all those who agreed to participate in the survey. Data collected were summarized with descriptive statistics; the normal distribution of the age data allowed central tendency to be summarized with the mean and the dispersion with ± standard deviation (SD). Data analysis used the Student t test, One-Way ANOVA, Pearson correlation analysis, Pearson Chi-Square test, and linear regression analysis. A p-value < 0.05 was considered statistically significant. We used the SPSS 22.0 statistical package program. (IBM Corp. Released 2013. IBM SPSS Statistics for Windows, Version 22.0. Armonk, NY: IBM Corp.).

## Results

### Participants

The mean age of the participants was 29.6 ± 8.0 years (min. 17 max. 55) with a gender distribution of 176 females (47.6%) and 194 (52.4%) males. Nurses were the most common profession (n = 162; 43.8%) followed by emergency medical technicians/paramedics (n = 124; 33.5%) and medical doctors (n = 84; 22.7%). 200 participants (54.1%) were single while 170 (45.9%) were married.

### Descriptive data

Less than half of the participants (48.5%; n = 173) felt competent in recognizing and managing violence against women, with nearly half of them (45.3%; n = 156) having encountered at least one VAW case within the last month. There was no significant difference between those who recently encountered and those who have not encountered VAW about feeling competent in its recognition and management (Chi-Square = 3.085; p = 0.079).

In case of encountering violence against women, 3.2% (n = 12) disregarded the abuse, 18.5% (n = 67) said they would try to reconcile the victim with the perpetrator, 68% (n = 246) informed the authorities, and 10.3% (n = 37) took some other actions.

### Outcome data

Fifty-seven participants (15.4%) answered ‘yes’ to the question ‘Would you like to rather be opposite gender?’ The wish of being of the opposite gender was significantly more among women (women: 27.8%; n = 49 vs. men: 4.1%; n = 8; Chi-Square = 39.831, p < 0.001).

Mean± SD of the VAW and sexist attitude scores were 18.9 ± 4.6 (min.15, max. 39) and 21.5 ± 5.6 (min. 12, max. 36), respectively. Among the VAW attitude questions, the highest agreement was with the item ‘The proverb “One who does not beats his daughter, will later beat his own chest” is correct’ (18.0% agree; n = 64) and the lowest agreement was with ‘A man may be forgiven if he was violent while drunk.’ (2.5% disagree; n = 9). The distribution of responses to each VAW attitude item together with the dimensions of the principal component analysis is given in Table 1.

**Table 1.** Attitudes towards violence against women among emergency health workers in Turkey.

	PCA Component				Agree		Neither agree nor disagree		Disagree	
Feelings	1	2	3	4	n	%	n	%	n	%
VAW is a public health problem.*				.780	8	2.2	37	10.1	320	87.7
VAW is disturbing to me.*				.758	11	3	21	5.8	332	91.2
I am disturbed by the news of women homicide in the media.*				.618	26	7.1	55	15.1	283	77.7
Justification (Physical Violence)										
Anger during a quarrel may be a reason why some women are beaten up.			.460		311	85.2	32	8.8	22	6.0
A man may be forgiven if he was violent while drunk.			.838		350	95.6	7	1.9	9	2.5
Women experiencing violence should NOT disclose the fact to others.			.539		302	83.4	29	8.0	31	8.6
There is no harm in slapping a women in some circumstances.			.516		313	86.0	30	8.2	21	5.8
Justification (Sexual violence)										
Provocative, low cut outfits are a cause of women being molested.	.763				222	60.8	82	22.5	61	16.7
It is the duty of a wife to have sex with her husband.	.517				291	80.6	35	9.7	35	9.7
A woman out of home late in the night should expect molestation.	.818				270	74.0	57	15.6	38	10.4
Sexual harassment is in part a woman’s own fault.	.685				303	83.9	43	11.9	15	4.2
General/cultural										
The proverb ‘One who does not beats his daughter will later beat his own chest’ is correct.		.578			185	52.0	107	30.1	64	18.0
Violence can be a solution sometimes.		.707			278	76.4	57	15.7	29	8.0
The proverb ‘A husband may love you and yet still beat you’ is correct		.664			311	85.9	24	6.6	27	7.5
There may be valid reasons for a man to beat up a woman.		.654			279	76.2	57	15.6	30	8.2

PCA = Principal Component Analysis; * = Reverse coded

The highest sexist response was to the item ‘It is the duty of the man to provide for his household.’ (37.3% agree; n = 136), while the lowest sexist response was with the item ‘A woman should earn less than her husband.’(13.7% agree; n = 50). The distribution of responses to each sexist attitude item together with the dimensions of the principal component analysis is given in Table 2.

**Table 2.** Attitudes towards sexism among emergency health workers in Turkey.

	PCA Component		Disagree		Neither agree nor disagree		Agree	
Family/Household	1	2	n	%	n	%	n	%
A woman should always be under the control of her man.	.683		206	57.4	100	27.9	53	14.8
The woman should be at home early, before her husband.	.657		166	45.6	98	26.9	100	27.5
The husband should be more educated than his wife.	.681		200	55.2	87	24.0	75	20.7
Child care is primarily a woman’s duty.	.597		86	23.6	151	41.5	127	34.9
Men should not do housework.	.640		236	64.5	74	20.2	56	15.3
Men have the duty to provide for their household.	.709		114	31.2	115	31.5	136	37.3
If a man does not approve, his woman should not work	.658		177	48.6	92	25.3	95	26.1
A woman should earn less than her husband.	.667		239	65.5	76	20.8	50	13.7
Work/Social Life								
Not all occupations are good for women.		.517	157	43.00	103	62.4	105	28.7
Before going out, a wife should first ask for permission from her husband.		.502	86	23.4	130	35.4	151	41.1
I believe in gender equity.*		.536	72	19.7	121	33.1	173	47.3
A woman should be free to spend her money as she wishes.*		.746	115	31.9	125	34.6	121	33.5

PCA = Principal Component Analysis; * = Reverse coded

Men had higher mean scores both on VAW attitudes (20.7 ± 5.2 vs. 16.9 ± 2.8; t = 7.927; p < 0.001) and on sexist attitudes (24.3 ± 5.3 vs. 18.6 ± 4.3; t = 1.714; p < 0.001). There were also marital status differences in the mean VAW attitude scores (married: 19.6 ± 4.9; single: 18.3 ± 4.3; t = 2.627; p = 0.009). However, there were no significant differences between the mean sexist attitude scores of married (21.7 ± 5.6) and single (21.4 ± 5.6) participants (t = 0.531; p = 0.596). Compared with non-exposed, those who were exposed to VAW within the last month had lower mean scores both on VAW attitudes (19.4 ± 5.2 vs. 18.2 ± 3.6; t = 2.278; p = 0.023) and on sexist attitudes (22.3 ± 5.6 vs. 20.8 ± 5.6; t = 2.320; p = 0.021).

There were striking opinion differences between women and men in some bivariate comparisons. While among women there were only two participants (1.2%) agreeing that there may be valid reasons for a man to beat up a woman, this figure was 28 (14.5%) among men. Also on some items querying sexist attitudes, such as ‘women coming home earlier, before their husbands,’ ‘taking permission to go out,’ ‘men doing housework,’ and ‘working of women,’ women and men differed significantly. These findings are presented in Table 3.

**Table 3.** Items with strikingly different opinions between women and men.

		Disagree		Neither agree nor disagree		Agree			
		n	%	n	%	n	%	Chi-Square	p
There may be valid reasons for a man to beat up a woman.	Women	161	93.1	10	5.8	2	1.2	52.241	< 0.001
	Men	118	61.1	47	24.4	28	14.5		
A woman should always be under the control of her man.	Women	135	78.9	30	17.5	6	3.5	66.946	< 0.001
	Men	71	37.8	70	37.2	47	25.0		
A woman should be back home early, before her husband.	Women	114	65.9	37	21.4	22	12.7	59.65	< 0.001
	Men	52	27.2	61	31.9	78	40.8		
Before going out, a wife should first ask for permission from her husband.	Women	66	38.2	68	39.3	39	22.5	59.165	< 0.001
	Men	20	10.3	62	32.0	112	57.7		
Men should not do housework.	Women	147	85.5	17	9.9	8	4.7	63.354	< 0.001
	Men	89	45.9	57	29.4	48	24.7		
If a man does not approve, his woman should not work	Women	119	70.0	32	18.8	19	11.2	62.433	< 0.001
	Men	58	29.9	60	30.9	76	39.2		

**Figure 1.**
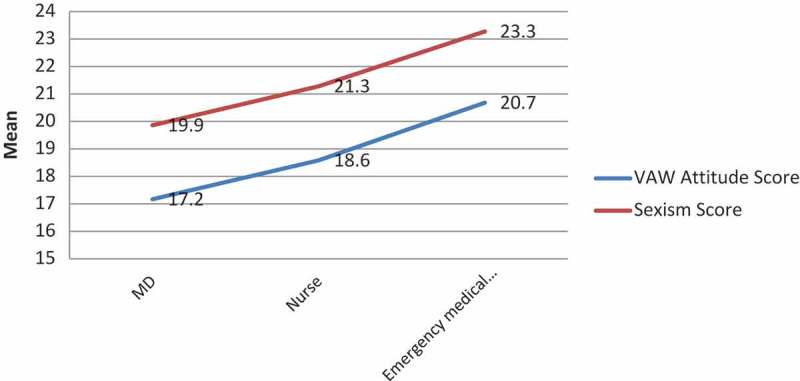
Distribution of the VAW attitude and sexism scores according to occupations.

There were also significant differences in the mean VAW and sexist attitude scores between different professions with medical doctors having the least scores (17.1 ± 2.7 and 19.8 ± 4.6 respectively), followed by nurses (18.5 ± 4.6 and 21.2 ± 5.7 respectively), and other staff (20.6 ± 5.2 and 23.7 ± 5.6 respectively) (ANOVA F; p for VAW and sexist attitude scores 14.934; < 0.001 and 9.272; < 0.001 respectively) (Figure 1).

**Figure 2.**
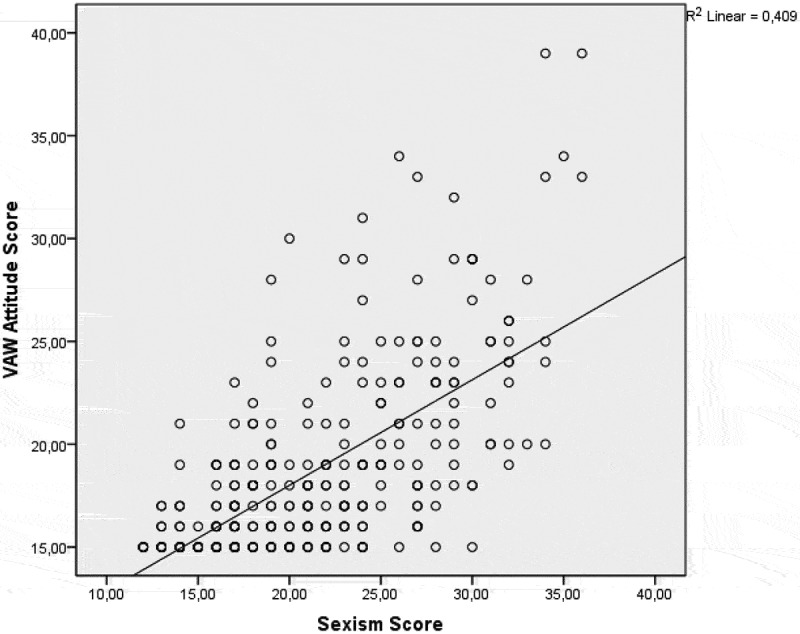
Correlation between VAW attitude scores and sexist attitude scores.

Pearson correlation analysis showed significant correlations between age and VAW attitude scores (r = 0.193; p < 0.001) as well as VAW attitude scores and sexist attitude scores (r = 0.639; p < 0.001) (Figure 2).

A linear regression model was built to check for the independent predictors of the VAW attitude score. Variables entered into the model were sexist attitude score, age, gender (dummy), and recent exposure to VAW (dummy). Age (B = 0.067; 95% CI: 0.014–0.119; p = 0.013) and sexism scores (B = 0.487; 95% CI: 0.407–0.566; p < 0.001) were independent significant predictors of the VAW attitude score. Exposure and gender were not significant in this model (p > 0.05)

## Discussion

### Key results

This study affirmed the connection between sexism and violence against women (VAW). We have empirical support for the idea that sexism is a significant correlate of acceptability of VAW. The attitudes against sexism and VAW are not encouraging, even in such a highly educated population like our study participants. Alarmingly, for health professionals who are expected to deal competently with emergency VAW cases, more than half of our study participants, did not feel competent. Although about half of the emergency service health workers encountered recent cases of ‎VAW, more than half of the surveyed did not see themselves adequately prepared to identify ‎cases of violence against women, nor to undertake necessary interventions.

### Interpretation

Confirming the results of Öztürk et al. [15], the majority of our study participants regarded violence against women as a public health problem. However, a mere sympathy for this issue does not necessarily translate into working skills, attitudes, and behaviors capable of dealing with the real problem in clinical settings. Several previous studies have shown that many health care workers in Turkey did not have an adequate education on violence against women [13,15–17].

Moreover, training alone in violence recognition may not suffice. A properly trained provider’s clinical ability to recognize a case of violence against women may not automatically trigger an appropriate helping behavior. This is especially true if the provider has a high tolerance and justification for violence, as suggested by an Istanbul study conducted on health personnel, in which 16.5% of participants regarded an open clothing style (‘low cut’) as a legitimate reason to abuse and sexually assault women [18].

Another, and perhaps even more worrisome layer of this problem is sometimes the very attitude of the victims, and/or of some female health care providers, who rationalize and condone violence against women. 37.4% of female participants in Karatas’s study [19] responded that ‘they approved to be beaten by their husbands if it was done out of a loving concern.’ In Arat and Altınay’s 2007 study in Turkey [20] 13.6% female participants agreed that ‘in some cases, men can beat their wives’ and thought that violence could be a legitimate way of solving a marital problem. This low rate may be attributed to a reduction over the years in the tolerance of violence, and explained by the higher level of education of the female participants in the Arat and Altinay study. Nevertheless, in our research in Erzurum, 18.5% of participating female health care personnel rationalized and condoned violence against women.

The attitudes of health professionals towards violence against women seem to point out a contradiction between the health professional’s cultural value orientation and norms, on the one hand, and the understanding of one’s scope of professional work, on the other hand. Items on traditional issues received higher responses supporting violence and sexism. The proverb ‘One who does not beat his daughter, will later beat his own chest’ and the phrase ‘It is the duty of the man to provide subsistence to his household’ are both rooted in the traditional Turkish culture. The fact that the Law for the Protection of the Family (Law 4320) in Turkey, dated ‎‎1998, is still not fully observed twenty years later suggests that legislative interventions may take a long time to be effective in Turkey.

One remedy to the sexist behaviors of men could lie in a positive exposure to women in their daily lives. Establishing an atmosphere of inter-gender respect and mutual empathy in discussions in workplaces, schools, and other public spaces could be helpful in this regard. The same suggestion applies for women as well. Intergroup contact-(positive) interactions with people from different social groups-is a strongly supported prejudice-reducing mechanism shown to reduce prejudice against a wide variety of outgroups [21]. Though, having found no significant difference in the sexist attitude scores in the married group implies that marriage alone as an interaction with the opposite sex may not suffice.

Although men in this well-educated population still bear sexist and violent attitudes towards women, female participants seem to be a step ahead in rejecting discrimination and violence. For our female population, there can be no valid reasons for a man to beat up a woman; this entails we could expect these women to resist violence. Dependence and subordination are the reasons given [22] for accepting the aggressive acts of men; these attitudes are not condoned by most educated women. In fact, education is a primary determinant of VAW, lower levels of schooling meaning more risk of exposure to violence. This argument may be used to explain occupational differences in the mean VAW and sexist attitude scores of our sample: While medical doctors have six years of post-high school training, the nurses have four, and emergency medical technicians and paramedics have two years of university education.

One study from Turkey indicated that among undergraduate students, gender, egalitarian gender roles and hostile sexism were variables predicting tolerance of verbal violence and attitudes towards divorce after physical violence; while gender and egalitarian gender roles were predictors of attitudes towards physical violence [23]. It was suggested that cultures of honor, such as the one in Turkey, prioritize defending individual and family reputations but in gender-specific ways, where men maintain honor via reputations for toughness, aggression, control over women, and avenging insults, while women maintain honor through obedience to men, sexual modesty, and religious piety [24]. As expected, it was demonstrated in a sample from Turkey that benevolent sexism positively predicted women’s honor beliefs, whereas hostile sexism positively predicted men’s honor beliefs. On the other hand, Islamic religiosity positively predicted honor beliefs for both genders [25]. Thus, it may be suggested that combating benevolent sexism and promoting feminist interpretations of Islamic religiosity may help and empower Turkish women to modify honor beliefs.

Sexism, as well as violence against women, is intermingled with many variables. Therefore, wherever possible, multivariate analyses should be sought in studying these problems. Gendered social norms and roles, changes in established gender norms and roles, men’s substance use, women’s separation from family, unemployment, rapid remarriages, forced marriages, and lack of enforcement in punishing abuses have all been shown to be significant factors in VAW [3,9,22,26–28]. Among the factors (sexist attitude score, age, sex, and recent exposure to VAW) we studied in the linear regression analysis, only age and sexism scores emerged as independent significant predictors of the VAW attitude scores. Thus, we advocate including the sexist attitudes in the study portfolio of VAW for future studies. These findings will perhaps contribute to answer the sociological question ‘Are the offenders sexist or just simply violent men?’ It was argued that men who assault women do not need to be sexist, they may be just violent individuals, who get away with their abuse easily because the society tolerates violence against women [29].

Extensive legal, political, as well as civil initiatives have been undertaken to combat VAW in Turkey. Over 200 women’s organizations contributed [30] to the drafting and adoption of the new Law 6248, which also gave significant power to police authorities to protect women at risk. Violence Prevention Following Centers (ŞÖNİM) were established under The General Directorate of Women’s Status [31]. Although the legal framework is largely in place, implementation lags behind in terms of capacity (number of shelters, trained staff) and infrastructure (e.g. coordination between policy and executive levels) [32]. Addressing the problem of domestic violence thus requires a concerted effort on the part of the authorities to not only invest in an adequate infrastructure to support victims, but also to raise public awareness and positively influence prevailing anachronistic mentalities about gender roles in society.

We had a particular reason to conduct this study in Erzurum. In addition to the patriarchal, conservative mentality explained earlier, Eastern Anatolia has a semi-feudal, traditional agricultural economy in which women do not have as many employment opportunities as they do in Western Turkey; consequently, women tend to be financially dependent on their husbands. Our findings, when compared with the 2002 study in Western Turkey [13], confirmed our assumption that although there is an improvement, medical emergency workers still have poor attitudes and behaviors towards violence against women. Aksan and Aksu had claimed that at least one reason to justify physical violence was accepted by 69.0% of female and 84.7% of male emergency department workers [13]. The findings are relevant as they imply a more acute need for education of health personnel, and policy changes to ensure that a mandatory continued medical education will be enforced.

### Limitations

One limitation of our study is that it does not include opinions of the general public. Comparing our findings with data from a representative general population would give much more useful data. Without a comparison group, it is difficult to know how much medical ‎emergency service workers differ from others. Additionally, as the survey did not include any measures of violence, we cannot claim that an acceptance of VAW would necessarily translate into a violent behavior. ‎

## Conclusion

An important conclusion of our study is that biased attitudes conducive to, or supportive of violence against women, were highly prevalent even in well-educated medical personnel who are responsible for responding with professional care to such cases. Alarmingly, while much more prevalent in men, those attitudes were also prevalent in women. There was an apparent relationship between sexist attitudes and attitudes condoning VAW. These findings suggest that educational and public health measures instituted to change such harmful attitudes must focus not only on violence per se, but equally so on sexism. To prevent gender discrimination

health care professionals need to reflect on their own gendered perspectives in clinical practice.

## Paper context

Although studies on the prevalence of domestic violence in Turkey abound, there is a knowledge gap on the first responders’ opinions on violence against women. Even less has been published on how sexism might influence health personnel’s attitudes. Our study examined the attitudes and behaviors of Erzurum medical emergency service workers towards violence against women and possible correlations with sexist opinions. Future public health measures must be focused not only on violence but also sexism.
